# The Iran Healthcare Professionals’ Charter of Rights

**DOI:** 10.34172/aim.34929

**Published:** 2026-01-01

**Authors:** Maryam Modabber, Alireza Parsapour, Vahid Moazzen, Hanieh Gholamnejad, Saeed Biroudian, Ehsan Shamsi Gooshki

**Affiliations:** ^1^Medical Ethics and History of Medicine Research Center, Tehran University of Medical Sciences, Tehran, Iran; ^2^Department of Public and International Law, Faculty of Law, Farabi Campus, University of Tehran, Tehran, Iran; ^3^Department of Medical Surgical Nursing, School of Nursing and Midwifery, Iran University of Medical Sciences, Tehran, Iran; ^4^Department of Medical Ethics, School of Medicine, Iran University of Medical Sciences, Tehran, Iran; ^5^Monash Bioethics Center, Monash University, Melbourne, Australia

**Keywords:** Charter of rights, Healthcare professionals, Iran, Medical rights, Professional ethics

## Abstract

**Background:**

In today’s rapidly changing social and cultural landscape, recognizing the rights of Healthcare Professionals (HCPs) alongside those of the patients has become essential for a fair and sustainable healthcare system. While patient rights are widely acknowledged, HCPs in Iran often face heavy workloads, poor work-life balance, and reduced professional dignity. These challenges not only affect their well-being but can also compromise the quality of care the patients receive. This report presents the development of the "Charter of Rights for HCPs of Iran," prepared by the Supreme Council of the Iranian Medical Council (IMC). The charter aims to create a clear framework that protects HCPs’ rights, promotes ethical practice, and supports the delivery of high-quality care nationwide.

**Development Process and Implementation:**

A comprehensive, consultative approach was used to develop the charter. The process began with a literature review, followed by semi-structured interviews with a diverse group of physicians, pharmacists, and dentists. Key themes were identified through content analysis, and the draft charter was refined based on feedback from focus groups and the Delphi method, ensuring its relevance, practicality, and acceptance by stakeholders.

**Key Findings:**

The charter includes six chapters and 59 articles addressing areas such as professional respect, workplace safety, fair compensation, continuing education, transparency, and professional autonomy. Each chapter outlines clear rights and responsibilities, highlighting the importance of balanced relationships between HCPs and patients.

**Discussion:**

The Iran HCPs’ Charter of Rights presents a comprehensive framework for safeguarding healthcare professionals’ rights, establishing multi-stakeholder accountability and addressing issues such as conscientious objection, aiming to mitigate systemic vulnerabilities and reduce dissatisfaction-driven turnover. This pioneering document offers a potential global model for balancing professional rights with quality patient care.

**Conclusion:**

Ratifying and implementing this charter represents a significant step toward recognizing and safeguarding the rights of HCPs in Iran. Its adoption is expected to enhance workforce well-being, strengthen ethical standards, and improve patient care. Future studies should assess the charter’s impact and uptake by key national stakeholders to guide further improvements.

## Introduction

 In recent decades, human and professional rights have gained global attention as essential pillars of sustainable development and social justice in healthcare. While patients’ rights have been progressively recognized and codified by governments and professional organizations internationally,^1^ developing documents that specify the rights of healthcare professionals (HCPs) has received comparatively less attention. Patient-centered care, emphasizing protection from harm, respect for autonomy, and equitable access to healthcare, illustrates this growing focus on recipients’ rights, yet a comprehensive framework addressing HCPs’ rights remains lacking.

 In early phases of modern medical ethics, patients’ rights often appeared in tension with the responsibilities of HCPs, who were traditionally viewed as the primary moral agents expected to fulfill the patients’ rights. The emergence of autonomy in contemporary bioethics challenged this paternalistic model, shifting power toward patients. Consequently, professional bodies such as medical and nursing councils have long prioritized safeguarding patients’ rights as their main mission to sustain the public trust toward the profession.^2,3^ However, the reductionist idea of natural confrontation between patients and HCPs’ rights has been increasingly oversimplified, as patients, while still potentially more vulnerable than HCPs, now exert greater influence, in both high-income countries and many low- and middle-income countries (LMICs). Patients’ voices are more echoed by civil society, legal, and regulatory systems. A good example of this change is seen in the UK. Reforms in the UK General Medical Council (GMC) after the 2007 *Trust, Assurance and Safety report*, shifted its structure from a profession-led model to one with equal representation of medical staff and laypersons, promoting greater transparency and public accountability.^4^ Globally, the rising number of legal complaints against HCPs further reflects this shift,^5,6^ prompting more defensive practices within the medical community.^7,8^ In addition, healthcare delivery has grown increasingly complex, involving governments, private institutions, pharmaceutical and medical device industries, and digital health technologies. HCPs, once seen as powerful actors, now operate within multifaceted systems shaped by political and financial agendas, rendering them vulnerable to exploitation, unacceptable and unsafe working conditions, armed conflicts, political pressures, violence, and bullying. Notably, incidents such as the murder of a cardiologist in Iran highlight extreme risks faced by HCPs.^9^

 Although newer perspectives on medical professionalism have begun to acknowledge the rights of physicians, earlier and prevailing views have largely overlooked these rights.^10^ This issue has led to problems in healthcare facilities, such as expectations to perform duties beyond their capabilities, lack of control over work schedules, harm to personal lives and the well-being of healthcare staff, and disregard for their rights.^11,12^ Since the rights of healthcare workers are not recognized, they often face unfair criticisms from individuals outside the field, which contributes to a heightened sense of inequality and violation of rights among physicians and healthcare staff.^13^ These inequalities affect the relationships between HCPs and recipients, potentially leading to a decline in service quality, increased stress and pressure on members of the medical community, and ultimately jeopardizing public health. Addressing HCPs’ rights is critical not only for their well-being but also for patient safety and the quality of care. Ethical frameworks should ensure a balanced respect for both HCPs’ and patients’ rights,^14^ fostering professional dignity, job security, and equitable healthcare delivery. In other words, order and justice (two interconnected dimensions of the same principle) should work together to achieve a fair balance between the rights and obligations of HCPs and recipients.

 In recent decades, Iran, like many other countries, has witnessed a growing movement toward recognizing patients’ rights. The Patients’ Rights Charter was first introduced in 2009 and later updated in 2014.^15,16^ Subsequently, in 2018, the Iran Medical Council (IMC) adopted its first Code of Ethics, requiring HCPs to respect patients’ rights, with noncompliance subject to disciplinary or professional sanctions.^17^ However, amid rapid social change, political tensions, economic sanctions, and waves of international migration of healthcare workers and brain drain, attention to HCPs’ own rights has remained limited. Physicians and other professionals often face systemic challenges including the exploitation of residents—linked to several reported suicides^18,19^; disrespectful treatment during medical complaint assessment processes; underpaid positions and lack of fair and permanent contracts; and the exploitation of junior doctors by more powerful professionals.^20^ For instance, so-called “ghost surgeons” may perform surgeries in place of well-known senior surgeons in private hospitals without being acknowledged, fairly compensated, or respected.^21^ Similarly, residents in university hospitals often carry out complex procedures without proper recognition or support. Other serious issues include acts of violence by patients and their families, compulsory service requirements without adequate financial compensation, and bullying or mistreatment by other HCPs.^22,23^ Recent data suggest that 82.1% of Iranian medical residents experience burnout.^24^ Additionally, 89% report exposure to workplace abuse,^25^ and 88.1% face workplace violence.^26^ There is also a worrying rise in depression, and suicide among Iranian medical residents, with around 20 suicide or attempted suicide cases reported in the past year—nearly 15 times higher than the national average.^27^ Collectively, these findings underscore the urgent need for structural reform.

 To address these challenges, the Supreme Council of the IMC developed the Iran HCPs**’** Charter of Rights, a comprehensive framework designed to protect the dignity, welfare, and professional autonomy of HCPs. Approved in 2022, the Charter, comprising six chapters and 59 articles, defines the rights and responsibilities of HCPs across domains such as respect, safety, education, transparency, and professional independence. While several international frameworks, such as the World Medical Association’s Declaration of Helsinki and Declaration of Geneva, have established ethical standards focusing on the protection of patients and research participants, few have comprehensively addressed the professional rights of HCPs.^28,29^ At the national level, countries such as the UK and Australia have adopted frameworks that indirectly protect healthcare professionals’ rights through broader human rights and employment laws. In the UK, the Human Rights Act 1998 has advanced a rights-based approach to healthcare delivery, promoting fairness, accountability, and professional responsibility.^30,31^ Similarly, in Australia, national regulatory reforms aim to balance professional accountability with equitable access to care within a unified legal and ethical framework.^32,33^

 In contrast, the Iran HCPs’ Charter of Rights explicitly codifies these rights within a single, legally recognized framework, addressing issues such as exploitation, workplace safety, and professional autonomy, thus representing a context-specific and pioneering model in global medical ethics and regulations.

## Development Process and Implementation

**Figure 1:**
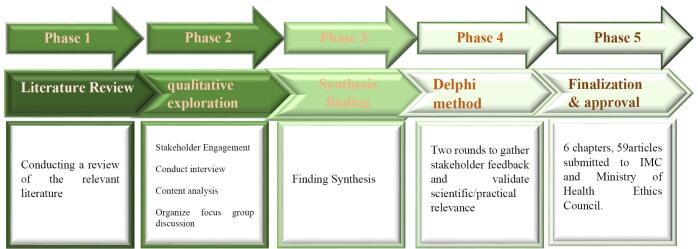
Methodological Framework for Developing the Iran HCPs’ Charter of Rights

 To develop a comprehensive Iran HCPs’ Charter of Rights, a mixed-method approach was employed in five phases: (1) a literature review to identify best practices and gaps; (2) qualitative interviews with purposively selected HCPs and key informants across Iran to capture perspectives on professional rights; (3) integration of findings from literature and interviews to draft the Charter; (4) refinement using a two-round Delphi method with experts and stakeholders; and (5) finalization and approval by the IMC Supreme Council and the Ministry of Health’s Supreme Ethics Council. Ethical approval was obtained, ensuring voluntary participation, confidentiality, and secure data handling (Figure 1).

 Findings from qualitative interviews with HCPs were organized into key themes, categories, and subcategories, as summarized in Supplementary File, Table S1. This evidence-based framework directly informed the development of the Charter, ensuring that each chapter and article reflects the priorities and concerns of HCPs. The Charter emphasizes that these rights should not conflict with patients’ rights or professional ethical guidelines and mandates the Council to advocate for these rights with relevant stakeholders while establishing a specialized advisory committee to oversee implementation.

## Iran’s HCPs’ Charter of Rights Content

 The Charter of the IMC underscores the critical importance of health as a fundamental value and a positive right of citizens, obligating governments to ensure equitable access to quality healthcare services. It recognizes the evolving complexity of health systems, where HCPs remain central advocates for patients’ rights despite the multifaceted nature of modern healthcare. The Charter addresses the historical emphasis on patients’ rights while highlighting the need to protect the rights of HCPs, noting that the rights of HCPs and recipients are interlinked. The IMC, guided by its legal mandate, aims to balance these rights through this Charter, which comprises 6 chapters and 59 articles (A full English translation of the Charter is provided in the supplementary file accompanying this article). It emphasizes that these rights should not conflict with patients’ rights or professional ethical guidelines, and it mandates the Council to advocate for these rights with relevant stakeholders while establishing a specialized advisory committee to oversee implementation.

## Chapter One: The Right to Professional Respect and Dignity

 This chapter outlines the right of HCPs to be treated with respect and dignity in all professional interactions, including with colleagues, patients, institutions, and media. It mandates the IMC to protect professionals from insult, humiliation, discrimination, or unfounded accusations, ensure privacy and confidentiality of professional information (except where legally required), and uphold respectful treatment during complaint investigations (Table 1).

**Table 1:** Summary of Chapter One: The Right to Professional Respect and Dignity.

**Article**	**Main Point**
1	HCPs have citizenship rights and the IMC must defend these through policy and legal interaction.
2	Professionals are entitled to respect, protected from insult, humiliation, or discrimination by all stakeholders.
3	Respectful behavior is required in interactions with the IMC’s staff and management.
4	Professionals deserve polite behavior during complaint investigations, with decisive action against violations.
5	Privacy must be respected, with restrictions on unauthorized audio/video recordings in healthcare settings.
6	Professional information must remain confidential, except for legally required disclosures.

## Chapter Two: The Right to Professional Security and Support

 This chapter focuses on ensuring professional, occupational, and social security for HCPs. The IMC is tasked with monitoring threats, providing psychological and legal support, and establishing mechanisms like emergency hotlines and ethical consultations. It emphasizes workplace safety, protection against violence and harassment, and fair treatment in legal proceedings, while addressing ethical dilemmas and reducing unnecessary summons (Table 2).

**Table 2:** Summary of Chapter Two: The Right to Professional Security and Support.

**Article **	**Main Point**
7	Professionals have the right to professional and social security, with the Council monitoring and planning improvements.
8	Access to counseling and emergency hotlines is required for incidents like harassment or violence.
9	Legal and advisory support must be provided to address rights violations.
10	Professionals are entitled to physical and social security in workplaces, especially in high-risk areas like emergency departments.
11‒12	Protection of privacy and personal safety during compulsory service assignments, including cultural orientation and training, and prompt institutional response and reporting in cases of serious workplace violence.
13	Protection against sexual harassment, with serious consequences for perpetrators
14‒15	Access to ethical consultation and minimal legal/regulatory concerns to reduce defensive medicine
16‒22	Fair, transparent, and non-discriminatory complaint procedures, including access to complaint details and written reasoning of decisions, dignified summons, sufficient opportunity for defense with legal and personal support, impartial review free of conflicts of interest, and safeguards against bias across specialties, seniority, or professional position.
23‒24	transparency in board membership, and use of professional licenses as collateral in legal cases
25‒28	Access to adequate professional liability insurance, fair and non-discriminatory access to job opportunities, and the right to practice within one’s verified training and competence without unjustified professional monopolies.

## Chapter Three: The Right to Equitable Access to Financial and Welfare Resources

 This chapter ensures that HCPs have access to fair income, welfare, and work-life balance. It mandates the IMC to monitor income levels, advocate for fair contracts, and ensure timely payments from insurance organizations. It also addresses equitable access to insurance, leave, protective equipment, and welfare facilities, particularly for trainees and those in high-risk settings (Table 3).

**Table 3:** Summary of Chapter Three: The Right to Equitable Access to Financial and Welfare Resources.

**Article**	**Main point**
29	Fair and sufficient income aligned with the length of training, level of expertise, workload, and professional responsibility, ensuring a dignified standard of living for all medical professionals, with particular attention to general practitioners and residents.
30‒31	Fair remuneration based on tariffs and protection from exploitative contracts or excessive working hours
32	Trainees have the right to appropriate remuneration for services provided.
33‒34	Inter- and intra-disciplinary justice in income, with transparent and non-discriminatory insurance contracts
35‒37	Timely payment of claims, freedom to choose workplace without unlawful obligations, and legal support for spouses to work and live together
38‒39	Access to protective equipment in high-risk situations, and adequate welfare, communication, and accommodation facilities
40‒42	Access to health/retirement insurance, emergency financial assistance, and fair leave/vacation benefits

## Chapter Four: The Right to Receive Necessary and Up-to-Date Training

 This chapter emphasizes the right to continuous, high-quality training for HCPs, both during studies and post-graduation. The IMC is responsible for ensuring adequate clinical supervision, up-to-date knowledge, and access to resources. It also addresses the need for clarity on professional boundaries and fair educational opportunities, while providing insights into common medical errors (Table 4).

**Table 4:** Summary of Chapter Four: The Right to Receive Necessary and Up-to-Date Training

**Article**	**Main point**
43	Adequate and supervised professional training throughout medical education, including high-quality clinical instruction and oversight, with ongoing monitoring to ensure scientific, ethical, legal, and communication competencies.
44‒45	Access to high-quality continuing professional development after graduation, and reliable access to up-to-date scientific resources (especially during emerging health crises) and infrastructure, including dependable internet.
46	Access to educational analyses of medical errors to reduce complaints
47‒48	Clarity on professional boundaries and fair access to educational/research opportunities

## Chapter Five: Transparency and the Right to Participate in Determining one’s Destiny

 This chapter ensures HCPs can participate in decision-making, express objections, and access transparent information about the IMC’s operations. It supports the right to form professional associations, participate in elections, and access financial/performance reports, fostering an inclusive and democratic professional environment (Table 5).

**Table 5:** Summary of Chapter Five: Transparency and the Right to Participate in Determining one's Destiny

**Article**	**Main point**
49	Right to express objections to laws/regulations harming professional or public interests
50‒51	Right to participate in health-related decision-making and free participation in professional elections
52	Access to transparent annual performance and financial reports of IMC
53	Right to strong professional and scientific associations, with Council support for their empowerment
54	Access to details of General Assembly and Supreme Council discussions, with confidentiality exceptions clearly justified

## Chapter Six: The Right to Independence in Professional Decision-Making

 This chapter affirms the right of HCPs to make independent decisions based on ethical, technical, and professional standards, free from external pressures. It allows refusal of services conflicting with personal beliefs (within legal and ethical limits), protection from coercion, and the ability to terminate harmful therapeutic relationships, ensuring professional autonomy (Table 6).

**Table 6:** Summary of Chapter Six: The Right to Independence in Professional Decision-Making

**Article**	**Main point**
55	Right to conscientious refusal of legally permitted medical services when they conflict with personal beliefs, provided patient safety is not compromised, within clearly defined and officially approved ethical and legal guidelines.
56	Protection of professional independence, especially for physicians working in high-pressure or restrictive institutional settings.
57	Right to refuse participation in criminal punishment without pressure or harassment
58	Ability to terminate therapeutic relationships for valid reasons, with proper documentation and patient support
59	Clinical decision-making free from external pressure, including protection against coercion to provide medically unjustified admissions, procedures, or services for non-clinical or financial motives

## Discussion

 The development and ratification of the Iran HCPs’ Charter of Rights by the IMC marks a groundbreaking milestone in the global discourse on HCPs’ rights. To our knowledge, this charter represents the first comprehensive regulatory document of its kind worldwide, explicitly addressing the professional, social, and ethical rights of HCPs in a structured and legally binding framework. By encompassing six chapters and 59 articles, the charter addresses critical domains such as professional respect, workplace security, financial equity, access to education, transparency, and decision-making autonomy (Table 7). Its inclusive scope extends beyond physicians to medical students, trainees, and allied health professionals, such as nurses, pharmacists, and dentists, thereby fostering a holistic approach to professional rights within Iran’s healthcare system.

**Table 7:** comperhensive Summary of the Charter of Rights for the HCPs of Iran

**Chapter**	**Article No.**	**Title of article**	**Key point**	**Main objective**	**Iran Medical Council’s Role**
Chapter I: The Right to Professional Respect and Dignity	1	Right to Respect as a Citizen and Professional	Respect and privacy	Protect professionals from insult, discrimination, and privacy breaches; ensure fair behavior in complaints	Monitor, policy-making, ensure confidentiality, and train staff.
	2	Right to Respect			
	3	Respect in Organization			
	4	Respect in Complaints			
	5	Privacy			
	6	Confidentiality of Information			
Chapter II: The Right to Professional Security and Support	7	Professional Security	Workplace safety and support	Provide psychological/legal support, ensure physical security, and streamline complaint processes	Establish hotlines, ethical consultations, and advocate for fair legal processes
	8	Psychological Counseling			
	9	Legal Counseling			
	10	Social Security			
	11	Security in Deployment			
	12	Reporting Violence			
	13	Protection from Harassment			
	14	Ethical Resources			
	15	Security in Professional Activities			
	16	Access to Complaint Mechanisms			
	17	Details of Complaints			
	18	Maintaining Professional Dignity in Summons			
	19	Right to Defense			
	20	Impartiality in Resolutions			
	21	Awareness of Rulings			
	22	Prohibition of Discrimination			
	23	Identification of Committee Members			
	24	Use of Practice License as Guarantee			
	25	Access to Professional Liability Insurance			
	26	Insurance for Trainees			
	27	Fair Job Opportunities			
	28	Right to perform authorized interventions			
Chapter III: The Right to Equitable Access to Financial and Welfare Resources	29	Adequate Income	Financial and welfare equity	Ensure fair income, work-life balance, and access to insurance/welfare facilities	Monitor income, advocate for fair contracts, and ensure timely payments
	30	Fair Wages			
	31	Work-Life Balance			
	32	Compensation for Trainees			
	33	Equity in Earnings			
	34	Documentation of Income Deductions			
	35	Timely Payment of Claims			
	36	Choice of Work Location			
	37	Family Support			
	38	Protective Equipment			
	39	Welfare Facilities			
	40	Access to Health and Retirement Insurance			
	41	Emergency Financial Assistance			
	42	Vacation and holidays			
Chapter IV: The Right to Receive Necessary and Up-to-Date Training	43	Necessary Education	Continuous education	Provide adequate training, access to knowledge, and clarity on professional roles	Monitor educational programs, provide in-service training, and resolve ambiguities.
	44	Continuing Education			
	45	Access to Updated Resources			
	46	Information on Complaints and Errors			
	47	Awareness of Permitted Interventions			
	48	Fair Educational Opportunities			
Chapter V: Transparency and the Right to Participate in Determining one's Destiny	49	Right to Protest	Democratic engagement	Enable participation in decision-making, transparent reporting, and professional associations	Facilitate protests, elections, and transparent communication
	50	Participation in Decision-Making			
	51	Participation in Elections			
	52	Access to Performance Reports			
	53	Professional Associations			
	54	Awareness of Official Meetings			
Chapter VI: The Right to Independence in Professional Decision-Making	55	Right to Refuse Service	Professional autonomy	Protect independent decision-making and freedom from coercion	Develop ethical guidelines and investigate violations of autonomy
	56	Professional Independence			
	57	Right to Refuse Participation in Punishment			
	58	Termination of Treatment Relationship			
	59	Independence in Decision-Making			

 Transparency is a cornerstone of the charter, woven into its fabric to ensure that HCPs are informed, empowered, and protected. Articles such as Article 52 (access to IMC’s annual performance and financial reports) and 54 (public disclosure of General Assembly and Supreme Council discussions) underscore a commitment to openness, enabling HCPs to engage meaningfully with the governance processes that shape their profession. This approach aligns with findings from Australian regulatory reforms, where greater transparency in complaint handling was associated with reduced practitioner distress and increased HCP satisfaction with the fairness of disciplinary outcomes^34^. This emphasis on transparency mitigates the historical opacity in professional regulation, fostering trust between HCPs, regulatory bodies, and the public. By mandating clear communication of complaint processes (Article 17) and decision rationales (Article 21), the charter ensures that HCPs are not left in the dark during disciplinary proceedings, reducing the risk of perceived injustice and enhancing procedural fairness. Research from high-income settings has similarly shown that transparent regulatory systems can significantly lower perceptions of inequitable treatment, including evidence that openly sharing decision-making processes can reduce complaint-related stress among physicians in the UK.^35,36^

 Participation and inclusion are central to the charter’s ethos, ensuring that HCPs have a voice in shaping their professional landscape. Articles 49–51 empower HCPs to engage in policy-making, protest unfair regulations, and participate in elections for professional bodies like the IMC. This participatory framework is particularly significant in Iran, where rapid social changes and economic sanctions have strained the healthcare system, exacerbating HCP vulnerabilities such as migration and burnout. A systematic review in LMICs showed that participatory approaches empower health workers and reduce stress and burnout, while also making interventions more effective by incorporating workers’ own insights; these approaches were linked to fewer interpersonal conflicts, better provider–client interactions, and substantial reductions in patient waiting times.^37^ Furthermore, by including medical students, trainees, and non-physician HCPs, the charter promotes an inclusive professional identity that transcends hierarchical and disciplinary boundaries. This inclusivity is critical in addressing issues like the exploitation of medical residents (Article 32) and ensuring equitable access to educational and professional opportunities (Article 48), thereby fostering a sense of collective agency and solidarity.

 Accountability is another pivotal principle, reframing the traditional patient–professional dichotomy. Rather than perpetuating a narrative that pits patient rights against HCP responsibilities, the charter adopts a systems-level perspective, holding multiple stakeholders (governments, healthcare institutions, insurance organizations, and regulatory bodies) accountable for safeguarding HCPs’ rights. For instance, Articles 9 and 15 demand robust legal and advisory support for HCPs facing complaints, while Article 38 mandates access to protective equipment during health crises. This approach acknowledges that HCPs operate within a complex ecosystem where their rights can be compromised by external pressures, such as economic exploitation (Article 30) or unsafe working conditions (Article 10). By emphasizing stakeholder accountability, the charter mitigates systemic vulnerabilities, such as those contributing to defensive medicine practices, which studies have linked to fear of unjust complaint assessments. Defensive medicine, characterized by excessive diagnostic or therapeutic interventions to avoid litigation, not only burdens healthcare systems but also undermines patient trust—a cycle the charter seeks to disrupt by fostering a fair and supportive professional environment.^7,8^ Beyond reducing defensive practice, evidence suggests that clear frameworks and strong institutional backing help clinicians feel professionally secure, more willing to report errors, and better supported throughout complaint processes. These structures do more than protect against legal anxiety; they foster a healthier patient safety culture, strengthen teamwork, and even reduce low-value care by easing the pressures that drive overly cautious medical decisions.^38-40^ Qualitative studies reinforce this view, showing that when clinicians work in environments where complaint pathways are transparent and institutions genuinely stand behind them, they experience less psychological strain and are far less inclined toward defensive medical practices.^41^

 A particularly innovative aspect of the charter is its approach to conscientious objection (Articles 55–57). By allowing HCPs to refuse certain interventions based on personal beliefs (provided that patient safety is not compromised), the charter strikes a delicate balance between individual autonomy and professional duty. In this context, establishing a clear and coherent framework is especially significant, as ambiguity in conscientious-objection processes has been shown to foster moral conflict and threatens healthcare professionals’ sense of integrity—conditions commonly associated with moral distress.^42^ The requirement for the IMC to develop a specific framework for conscientious objection (Article 55) is a forward-thinking mechanism that ensures ethical consistency while respecting diversity of thought. This provision sets a global precedent, as few regulatory frameworks explicitly address conscientious objection in such a structured manner, offering a model for other nations grappling with similar ethical dilemmas.

 The charter’s implications extend beyond Iran, offering a blueprint for global healthcare systems seeking to balance the rights of HCPs and patients. Global surveys confirm that workplace violence compromises the quality of care and places healthcare provision at risk, directly affecting patient safety.^43^ Moreover, LMICs are estimated to lose US$15.86 billion annually from physician migration alone, with India, Nigeria, Pakistan, and South Africa incurring the greatest total costs.^44^ Iran similarly experiences substantial physician migration, with an estimated $50-70 billion annual loss from scientific professional emigration^45^, exacerbated by economic sanctions and compensation inequities. These losses include the substantial economic impact of investing in the training of a health workforce that subsequently leaves. By addressing systemic issues such as workplace violence (Article 13), economic exploitation (Article 30), and lack of professional autonomy (Article 59), the charter tackles the root causes of HCP dissatisfaction and turnover, all of which have been linked to diminished quality of patient care. Additionally, its emphasis on continuous education (Articles 43–45) and access to up-to-date resources ensures that HCPs remain equipped to deliver high-quality care in an ever-evolving medical landscape.

## Conclusion

 The Iran HCPs’ Charter of Rights is a pioneering effort to elevate the professional dignity, security, and well-being of HCPs while enhancing patients’ health through a balanced and just healthcare system. Its principles of transparency, accountability, and inclusion provide a robust framework for addressing longstanding imbalances in the patient–professional relationship. By safeguarding HCPs from exploitation, violence, and undue regulatory pressures, the charter not only strengthens the resilience of Iran’s healthcare workforce but also sets a global standard for professional rights advocacy. Future research should focus on evaluating the charter’s implementation, its impact on HCP retention and patient care quality, and its potential adaptability in other socio-political contexts. The IMC’s commitment to monitoring and refining this framework will be critical to its sustained success, ensuring that it remains a living document responsive to the evolving needs of Iran’s healthcare community. Further studies for assessing the influence of this document on Iran’s healthcare system, including its endorsement by main stakeholders, are essential next steps.

## Limitation

 A notable limitation is lack of public consultation, limiting the incorporation of wider societal viewpoints in the charter’s development.

## Supplementary File

A full English translation of “ The Iran Healthcare Professionals’ Charter of Rights “ is provided as a supplementary file to this article.
