# Supplementary material for: The Iran Healthcare Professionals’ Charter of Rights
Source: Arch Iran Med. 2026 Jan 1;29(1):44–52. doi: 10.34172/aim.34929 (PMC13338497; doi:10.34172/aim.34929)
Supplement: Supplementary file 1 — A full English translation of “ The Iran Healthcare Professionals’ Charter of Rights “ is provided as a supplementary file to this article. [file aim-29-44-s001.pdf]

Arch Iran Med. January 2026;29(1):44-S1
doi:10.34172/aim.34929

http://journalaim.com

The Iran Healthcare Professionals' Charter of Rights

Introduction

Throughout history, "health" has always been the focus of special attention as a divine blessing and a central value. This attention is more tangible in today's world than ever before; such that now, it is recognized as a self-evident duty for governments to ensure the provision of health services to citizens. This means that although in the past, the "right to health" was largely recognized in the sense of freedom to receive health services for individuals, today it is considered a positive right of citizens; a right, based on which the government has the duty to either provide a decent and acceptable level of health services directly or to ensure the provision of the desired level of these services by designing specific mechanisms. In the meantime, a government is considered more successful if it can provide access to a more desirable level of health services for all and in an equitable manner. Unlike past centuries when doctors and some other Healthcare Professionals (HCPs) were the only main players in this important field, today's health systems include many components with complex relationships, and the provision of desirable health services is the result of their proper interaction. However, given the important role of social factors in health and disease incidence, health professionals, as the most important pillar of these multi-layered and complex systems, are, more than ever, the main defenders of the rights of patients and other recipients of health services.

In recent decades, following widespread social and cultural changes worldwide, the issue of respecting patients' rights has been in the spotlight for various reasons. In many countries, the rights of citizens as recipients of health services have been codified in documents with the general title of "Patient Bill of Rights." This is while not much attention has been paid to respecting the rights of HCPs, to the extent that, with the assumption that HCPs have more power than service recipients and that patients and other health service recipients are the more vulnerable party in this relationship, there is a kind of hesitation and reluctance in raising the issue of HCPs' rights. However, the relationship between these two groups is so intertwined and deep-rooted that respecting the rights of one requires respecting the rights of the other, and a unilateral approach to patients' rights not only fails to solve any problems, but also deepens the gaps. On the other hand, the idea that the rights of these two groups are in conflict with each other is wrong. In many cases, the authority to enforce the rights of HCPs and recipients is shared. Although the addressee of the obligation to respect the rights of health care recipients is, in many cases, the service providers, or in other words, members of the HCPs, the reverse is not true; Because, in most cases, respecting the rights of the HCPs is the responsibility of governments, institutions, organizations, companies, and other stakeholders; of course, respecting a small part of these rights is also the responsibility of the people, as recipients of health services.

In this context, the Medical Council of the Islamic Republic of Iran has tried to prioritize the rights of patients over the decades of its activity in order to achieve its inherent goals and duties, as stated in the Law on the Establishment of the Medical Council of the Islamic Republic of Iran and other upstream laws that include appropriate and proportionate protection of the rights of recipients and HCPs of health services. In this process, in 2018, the "Code of Ethics for Members of the Iran Medical Council," which includes the duties of members of the Iran Medical Council and the HCPs to effectively respect patients' rights and maintain and promote mutual trust between HCPs and recipients, was approved by the Supreme Council of the Medical Council. Now, in order to maximally respect the rights of patients and recipients of health services on the one hand, and the rights of members of the HCPs, as providers of health services, on the other, the "Charter of Rights of the HCPs " in six chapters and 58 articles was

approved by the Supreme Council of the Iran Medical Council on the 14th of Mordad 1400 (August 5, 2021)

The Supreme Council of the Iran Medical Council emphasizes that the rights contained in this charter should not, under any circumstances, be interpreted in conflict with the rights of recipients of health services contained in the "Patient Rights Charter", as well as the duties contained in the " Code of Ethics for Members of the Iran Medical Council". In cases where the authority for exercising the rights of members of the HCPs is the medical Council itself and its subordinate bodies and departments, the provisions of this document are effective and binding from the date of approval. In cases where the addressees of this document are other institutions or organizations, the Director General of the Medical Council must prioritize and follow up on the provisions of this document as the main strategy in dealing with the relevant institutions. It is necessary for all organs of the Iran Medical Council, in coordination and cooperation with other professional institutions, especially the Medical Scientific Associations, to demand that all stakeholders observe the rights contained in this charter. In order to create the necessary grounds for the implementation of the provisions of this document and to follow up and monitor its proper implementation, it is necessary to form a specialized advisory commission under the Supreme Council of the Medical Council. The specialized-advisory commissions on medical ethics and the plans and bills will jointly propose the details of the formation and operation of the said commissions, and the Supreme Council of the Medical Council will approve them. Members of the HCPs in this document include HCPs and those affiliated with the subject of Article 1 of the Disciplinary Regulations of the members of the Medical Council of the Islamic Republic of Iran; learners, students, and interns, especially those who are also official members of the Medical Council during their learning period, as well as assistants in specialized and post-specialized courses in various fields, are also among them.

Chapter One: The Right to Professional Respect and Dignity

Article 1. Members of the HCPs, like other members of society, enjoy citizenship rights in various professional, social, cultural and political aspects and pursue the fulfillment of these rights within the framework of the law and mutual social responsibility. The Iran Medical Council is obligated to defend the citizenship rights of members of the HCPs by continuously assessing the situation, making appropriate policies and planning, and interacting with relevant legal authorities.

Article 2. Members of the HCPs have the right to respect commensurate with their dignity and profession. Accordingly, members of the profession should be protected, as far as possible, from any treatment that involves or entails insult, humiliation, stigma, labeling, unfounded accusations and discrimination. This right includes respect from colleagues, patients and companions, managers of health care institutions, policymakers, the media and other stakeholders. Accordingly, medical students have the right to be respected by their professors, senior colleagues, and other colleagues and staff of health care centers. The Iran Medical Council is obligated to always be vigilant at a macro level to protect the privacy of the profession and, if any systematic disrespect is observed, to investigate the roots and causes of such cases and provide appropriate feedback and response.

Article 3. Members of the HCPs have the right to be treated with respect commensurate with their dignity and profession in their face-to-face and offline contacts with the Iran Medical Council. All managers and staff of the Iran Medical Council are required to constantly monitor the behavior of the organization's employees, ensure compliance with this issue, and provide the necessary training to the organization's employees.

Article 4. Members of the HCPs have the right to experience polite treatment, appropriate to medical ethics, and free from any insult, humiliation, unfounded accusations, and stigmatization during the handling of complaints related to their professional activities that have been filed against them with the relevant authorities. If any of the individuals involved in the investigation process, whether employees

or members of the Medical Council's disciplinary boards, deviate from this framework, it is necessary to deal with them quickly and decisively in a deterrent and serious manner. The Iran Medical Council is obligated to follow up and demand the exercise of this right from other investigating institutions, while implementing these cases in internal investigations.

Article 5. Members of the HCPs have the right to have their privacy fully respected by all stakeholders, including recipients of health services, and to feel secure in their relationships with patients. Any audio or video recording of the stages of health service provision by any of the stakeholders, including patients and recipients of health services, as well as institution officials, will be possible only after obtaining permission from those members of the HCPs who are exposed to such interventions and whose privacy is at risk. Recording images in public spaces of health care centers, if done in accordance with current regulations and within the framework of the law and publicly announced, is outside the scope of this article.

Article 6. Members of the HCPs have the right to demand that all their professional information be kept strictly confidential, including personal information, information related to judicial or law enforcement cases related to professional activity, financial information, and other information that, due to their membership in the profession, is made available to various authorities and stakeholders, including the Iran Medical Council, the Forensic Medicine Organization, medical universities, and institutions where they work and, except as provided by law, it shall not be made available to any third party, whether natural or legal, without their permission. Any publication or use of information of members of the profession, in a manner that identifies individuals, is possible only after obtaining the express permission of the owner of the information. It is clear that the use of information that has been irreversibly anonymized is permissible within the framework of the regulations. This right does not include matters that are part of the inherent and legal duties of the Iran Medical Council, such as membership of individuals in the Medical Council, the city and place of work of members, and other matters that are legally required to be reported. History of disciplinary and professional convictions can be reported if there is a legal basis or requirement.

Chapter Two: The Right to Professional Security and Support

Article 7. Members of the HCPs have the right to the necessary support for professional, occupational and social security. The Iran Medical Council is obligated to continuously monitor the status of professional security of members of the HCPs using various tools and, while identifying threats, opportunities, strengths and weaknesses, make appropriate plans to improve the level of professional security of its members. The Supreme Council of the Iran Medical Council recognizes the expectations of members of the HCPs from the legislative, executive, and judicial branches to approve and implement the necessary laws and regulations to create professional security in various dimensions, and considers the Iran Medical Council obligated to pursue this logical demand.

Article 8. Members of the HCPs have the right to access the Medical Council’s counseling and psychological support system following injuries resulting from incidents such as harassment or violence. The Medical Council’s organization is required to establish a national emergency hotline for specific cases so that members of the HCPs can immediately contact the Medical Council or the region in question and request assistance if needed.

Article 9. Members of the HCPs have the right to access the legal and advisory system and support within the Iran Medical Council to address violations of their rights as set forth in this document. The Iran Medical Council is required to establish an appropriate mechanism to guide members of the HCPs and provide access to legal advisory services. At the same time, the Iran Medical Council is obligated to defend with all its might the professional and personal security and dignity of any member of the profession, in cases where it is determined that any member of the profession has been subject to

questioning or judicial and administrative consequences, or the like, for any reason, due to or as a result of performing their professional duties, which they have performed correctly and in accordance with scientific, technical, and ethical standards.

Article 10. Members of the HCPs have the right to an appropriate level of social and physical security and professional dignity in their workplace; therefore, it is justified and affirmative for members of the HCPs to expect that health care institutions, especially hospitals, clinics, dispensaries, and the like, make necessary provisions for physical protection against possible harm. Ensuring physical security in the emergency department is also of particular importance.

Article 11. In cases where members of the HCPs are sent to different parts of the country to fulfill legal obligations, the sending institution is responsible for protecting their privacy and security. If there are working conditions and specific cultural or local sensitivities, including regarding the culture, customs, traditions and habits of the people, the ignorance of which by members of the HCPs may disrupt their security and peace or lead to a violation of the rights of the patient, the aforementioned institution is expected to take action to prepare content and provide appropriate training in this regard. The Iran Medical Council, both at the national and local levels, is obligated to follow up on the fulfillment of this task by other institutions and directly provide the necessary training, especially to those of its members who are starting their work in a region.

Article 12. In cases where serious violence occurs against any member of the HCPs in the environment of professional activity or related to professional activity, which results in serious physical injury, it is necessary for the local Medical Council to submit a report to the Director General of the Organization as soon as possible and within a maximum of ten working days, while carefully examining the matter and conducting a field visit, if necessary.

Article 13. Members of the HCPs have the right to be free from any form of sexual violence, assault, or harassment in their workplace. Any behavior or speech that is explicit, metaphorical, simile, or implied, and that is expressed or done outside the scope of professional necessity and that causes humiliation, insult, intimidation, or psychological or physical harm to the recipient, is considered sexual harassment. The expectation of members of the HCPs from all relevant stakeholders and officials, including officials of medical institutions, to prevent, not conceal, and deal seriously and deterrently with any type of sexual assault and harassment is reasonable and approved by the Iran Medical Council. If members of the profession are exposed to violence or sexual harassment by other colleagues in the HCPs and the institutions or members inform the Iran Medical Council, in addition to other legal actions, this act must be considered serious as unprofessional behavior, and the provincial Medical Ethics Expert Commission must review it out of turn, and the law enforcement agencies must immediately and out of turn follow it up until a result is reached.

Article 14. Given that one of the situations that cause tension among members of the HCPs is cases of ethical tension resulting from facing ethical dilemmas and questions, members of the HCPs have the right to the necessary resources and guides available in such situations. Since resolving such issues is sometimes complex and existing guidelines alone cannot provide answers, the Iran Medical Council is required to establish a consultation mechanism to answer such questions and help confront ethical dilemmas, and to inform members of the profession. The Supreme Council of the Iran Medical Council recognizes the right of access to professional ethics advice for members of the profession and, given the existential nature of the Iran Medical Council, considers the organization obligated to provide this service to its members.

Article 15. Members of the HCPs have the right to operate in an environment with minimal concern about the legal and regulatory consequences arising from their professional status. In this regard, it is appropriate for all institutions involved in the process of handling complaints received from members of the HCPs to consider the consequences of making the professional environment of members of the

HCPs unsafe, including the growth of defensive medicine and the reduction of the productivity of the health system. The Supreme Council of the Medical Council confirms the expectations of members of the HCPs from these institutions to prevent the repeated summons of professionals to judicial and law enforcement institutions, shorten the process of handling submitted cases, and prevent delays in processing and proceedings. In this regard, it is necessary for the Iran Medical Council to strive to establish complete coordination between all institutions handling complaints received, including the courts, the Forensic Medicine Organization, and the Iran Medical Council, and to minimize repeated visits by members of the HCPs.

Article 16. Members of the HCPs have the right to an efficient system for handling medical complaints. The Iran Medical Council is obligated to fully consider the rights of patients and other recipients of health services, and to propose and pursue appropriate mechanisms and solutions, such as establishing an amicable arbitration system or leveling the complaint handling system to prevent, if possible, unnecessary and repeated summons of members of the profession, which will ultimately cause harm and damage to recipients of health services. These mechanisms should not prevent patients from pursuing their rights.

Article 17. Members of the HCPs have the right, upon receiving any summons from institutions handling complaints related to professional activity, to receive, along with the summons, the details of the complaint to the extent legally possible, so that they can examine the matter before referring it and attend the meeting with greater preparation. The Iran Medical Council is obligated to pursue the exercise of this right from all relevant authorities.

Article 18. Any summons of members of the HCPs to institutions handling complaints, including judicial and law enforcement authorities, should be in a manner that does not damage the professional dignity of the person being summoned. The Iran Medical Council is required to anticipate and implement a specific mechanism for the implementation of this article in proceedings conducted by the Medical Council's law enforcement area, while pursuing this issue with other institutions.

Article 19. Members of the HCPs have the right to adequate opportunity to defend themselves in the process of investigating complaints raised against them. In this regard, it is appropriate for the investigating institutions to provide the necessary resources to ensure the presence and effective defense of members of the HCPs. Members of the HCPs should be able to bring at least one trusted person with them to the hearings and benefit from the services of their legal counsel. In handling complaints in the Iran Medical Council, members of the profession must be able to have the opportunity to defend themselves at all stages of the proceedings in the primary, appeal, and supreme boards. The investigating unit in the organization must also provide all the necessary documents required to prepare a defense, within the framework of the regulations, to the organization member. The Iran Medical Council is obligated to follow up and demand the exercise of this right from other investigating institutions, while implementing these matters in internal investigations.

Article 20. Members of the HCPs have the right to an impartial and fair system for handling complaints. No person involved in the handling process should have a material conflict of interest. In this regard, the Iran Medical Council is obligated to ensure this at all stages, including before the start of the hearings of the hearing panels. Any member of the board who has a material conflict of interest with any of the parties should be excluded from participating in that decision-making. In addition to implementing these measures in internal reviews, the Iran Medical Council should pursue and demand that other review bodies exercise this right. If it is later determined that these conditions were not met in the review, the ruling issued will be tainted and will need to be reviewed.

Article 21. Members of the HCPs have the right to be informed of the reasons and arguments for the decisions made against them during the examination of complaints related to their professional activities submitted to the relevant authorities. It is not sufficient to be informed of the final decision alone and it

is necessary to inform the interested professional, in writing, of the reasons for the decision. In addition to implementing these measures in internal investigations, the Iran Medical Council must pursue and demand the exercise of this right from other investigating institutions.

Article 22. Members of the HCPs have the right not to be discriminated against in the process of handling complaints against them. Given that in many pending cases more than one member of the profession is involved, it is necessary to make every effort to ensure that justice is fully observed between the professionals involved. Any discrimination based on specialization, work experience, job position, etc. is prohibited. It is necessary to design a mechanism within the framework of the regulations so that in cases where there is a possibility of bias or unfair decision due to reasons such as the managerial and professional position of one of the interested professionals, for example, in the management hierarchy of the regional Iran Medical Council, the review process is entrusted to an impartial group. In addition to implementing these measures in internal investigations, the Iran Medical Council must also pursue and demand the exercise of this right from other investigating institutions.

Article 23. Members of the HCPs have the right to know the members of all boards involved in the process of handling complaints against them. The membership of individuals in these boards must be informed to members of the profession, and the names, specialties, and professional and occupational positions of individuals must be available to members of the profession. If members of the profession allege a conflict of interest or evidence of a potential violation of impartiality regarding any member of the board, the reasons must be presented to the relevant official and, if the arguments are found to be valid, the person in question must be removed from the review process. In addition to implementing these measures in internal reviews, the Iran Medical Council must pursue and demand this issue from other review bodies.

Article 24. Members of the HCPs have the right to use their practice license or civil liability insurance as collateral in court, if necessary, during the process of handling cases filed against them. The Iran Medical Council is obligated to pursue and demand the implementation of this issue from the judiciary.

Article 25. Members of the HCPs have the right to access appropriate, diversified and adequate professional liability insurance, commensurate with the discipline. Such insurance must be sufficient to cover their civil liability and its obligations shall not be subject to the passage of time. On the other hand, the conditions stated in the insurance policy should not contain content that is contrary to professional ethics, such as requiring professionals not to disclose medical errors to the patient. The Iran Medical Council is required to amend professional liability insurance contracts to improve their content.

Article 26. The provision of professional liability insurance for specialized and subspecialized residents and other categories of learners who, while being learners, are also expected to have professional legal liability is the responsibility of the hospital or university where they study, and the Iran Medical Council is obligated to pursue and demand the availability of this insurance for all individuals in need.

Article 27. All members of the HCPs have the right to fair employment opportunities. The Iran Medical Council, while identifying areas of discrimination in access to employment opportunities, must take steps, in cooperation with various stakeholders, to eliminate areas and instances of discrimination.

Article 28. Members of the HCPs have the right to perform medical interventions within the framework of laws and regulations in areas where they have received appropriate training and acquired the necessary professional competence. The Iran Medical Council is obligated to plan and take action to increase the public's access to standard and quality services, using all available capacities, including the capacity of the National Commission for Professional Qualifications, while preventing the activities of individuals without professional qualifications, to abolish any unjustified and unjustified monopoly in the provision of health services by members of the HCPs and to prevent the creation of such monopolies.

Chapter Three: The Right to Equitable Access to Financial and Welfare Resources

Article 29. HCPs have the right to an income sufficient to enable them to lead a dignified life. The level of income of members of the HCPs should be proportionate to factors such as the long period of education, the extensive abilities required for the profession, the difficulty of the work and the great responsibility of the individuals during their employment. The sensitivity of the medical field requires highly capable individuals to enter this field; therefore, lack of a clear and accessible horizon for those working in these professions to enjoy a desirable life will be detrimental to society and the people in the long run. The Iran Medical Council is obligated to examine the conditions and strive to create appropriate and adequate financial and economic conditions and a dignified life for all those working in the medical professions. In this context, it is very important to pay attention to certain groups, including general practitioners and residents. The Iran Medical Council is required to periodically monitor the average income of various medical groups and report to the Supreme Council of the Medical Council.

Article 30. Members of the HCPs have the right to receive remuneration for their professional activities, based on approved tariffs and fair contracts. Members of the Iran Medical Council have the right to be protected from any form of economic exploitation, including unilateral and unfair contracts. The Iran Medical Council is obligated to empower members of the profession to defend this right, and to propose and support a proportionate and fair framework for such contracts by reviewing relevant contracts, including contracts concluded with insurance companies or contracts between professionals and institutions.

Article 31. Members of the HCPs have the right to a reasonable work-life balance. All members of the profession, including professionals employed by institutions or organizations, as well as residents in specialized and subspecialty fields, have the right to work standard working hours. Any imposition of working hours in excess of the standard hours on members of the profession or of working hours in excess of the approved educational program on learners of these fields is an example of exploitation and is unjustified. The Iran Medical Council is obligated to defend this right of professionals, residents, and other learners who are members of the organization.

Article 32. Clinical trainees in medical sciences, including specialty and subspecialty residents, have the right to receive appropriate remuneration for the interventions and services they provide to health care recipients. A portion of the remuneration for the services they provide should be paid appropriately to the service provider. The Iran Medical Council is obligated to provide a fair framework for paying trainees' fees, including specialized and subspecialized residents, by pursuing the issue with all relevant institutions, especially the Ministry of Health and Medical Education.

Article 33. Members of the HCPs have the right to enjoy inter- and intra-disciplinary justice in the amount they receive. In order to establish inter- and intra-disciplinary justice among medical professionals, the Iran Medical Council needs to propose clear criteria for determining and leveling health service tariffs in order to eliminate discrimination among professionals, in a way that members of the profession feel that they are being treated fairly. The process of determining the relative value of services should be explained to members of the profession in a transparent manner, and those involved in the process of determining the relative value of services should not have any effective conflict of interest.

Article 34. Any action by insurance organizations or medical institutions to deduct from the income of members of the HCPs must be documented and accompanied by the presentation of sufficient evidence. At the same time, the conclusion of private and public insurance contracts with members of the HCPs must be based on clear and announced criteria and free from any discrimination or non-transparent

relationships. The Iran Medical Council is obligated to communicate this procedure transparently to members of the HCPs by interacting with insurance organizations.

Article 35. Members of the HCPs have the right to receive their claims from insurance organizations and medical institutions as soon as possible, and to receive compensation for any losses and damages due to delays in payment. The Iran Medical Council is obligated to pave the way for its members to exercise this right by examining the legal grounds.

Article 36. Members of the HCPs, like all other professions, have the right to choose the place of their professional activity based on their personal preferences and life circumstances. Since any restriction on legitimate freedoms is only permissible by law, it is not acceptable to impose extra-legal obligations on applicants or successful candidates for various examinations, including the specialist and subspecialist assistant examinations. Given the possible negative impact of such obligations on the physical, mental, and social health of members of the profession, the Iran Medical Council is obligated to continuously review such obligations and, in cases where the obligations appear unfair, to seek legal avenues to rectify the situation and, if necessary, amend the laws.

Article 37. Members of the HCPs have the right to be protected by laws and regulations that allow married couples to live together while changing their place of employment for the protection of the family. Since this right, as a right of citizenship, applies to all members of society, The Iran Medical Council is obligated to examine the legal grounds and pave the way for its members to exercise this right. Cases where an individual's commitment to serve in a geographical area is the basis for his or her enjoyment of a special privilege, such as a quota for admission to a specific field or location, are not included in this article.

Article 38. In times such as infectious disease outbreaks, members of the HCPs who, due to the nature of their work, are exposed to increased risks in the course of providing health services to patients, have the right to access the necessary protective equipment to reduce the risks to a reasonable and acceptable level. Health service providers, such as hospitals, are obligated to provide and guarantee this access. The Iran Medical Council must also follow up on the fulfillment of this right.

Article 39. Members of the HCPs, especially residents and trainees, have the right to access necessary and appropriate welfare and communication facilities at the place where they provide their services. The provision of these welfare facilities is the responsibility of the institution where the services are provided. The Iran Medical Council is required to develop and implement the desired welfare standards for the boarding of students in teaching hospitals, the boarding of members of the HCPs sent to complete the legal obligations plan, hospital doctors on duty, and the like, in cooperation with relevant institutions, especially the Ministry of Health and Medical Education. It is also necessary to focus on creating appropriate opportunities for the education of children of professionals, alongside local students, through various methods.

Article 40. Members of the HCPs have the right to access adequate health insurance and retirement services. This is particularly important for those members of the HCPs who are not permanently employed by institutions and organizations. The Iran Medical Council is obligated to ensure the payment of insurance premiums for members of the HCPs who cooperate with medical institutions under temporary and short-term contracts, as well as for trainees who are members of the organization, especially specialized and subspecialized assistants, based on the labor law and other laws of the country, and, if possible, to provide the necessary infrastructure to establish an appropriate retirement and medical insurance system for members. The Iran Medical Council must regularly monitor the insurance status of members of the profession and report to the Supreme Council of the Medical Council.

Article 41. Members of the HCPs have the right to access, to the extent possible, an emergency assistance or loan system. The Iran Medical Council is required to establish an appropriate mechanism

for this issue through the Cooperative and Welfare Fund so that it can provide financial assistance to members of the HCPs in the form of loans or grants, when necessary, to meet their essential needs.

Article 42. All members of the HCPs , including trainees, have the right to enjoy leave and vacation benefits; therefore, when a doctor or treating professional intends to use their leave, the Iran Medical Council must, in order to protect the rights of patients to continue receiving health services, prepare and propose standards for transferring the service delivery process to a qualified and preferably equal professional, and also follow up with the relevant authorities to provide the necessary opportunity for this issue.

Chapter Four: The Right to Receive Necessary and Up-to-Date Training

Article 43. Medical students and members of the HCPs have the right to receive the training necessary to fulfill their professional responsibilities during their studies. In this context, receiving appropriate clinical training, especially adequate supervision by professors of the performance of clinical interventions by students, especially specialty and subspecialty residents, is very important. The Iran Medical Council is obligated to ensure the adequacy of this training in various scientific, technical, and social dimensions, as well as in the areas of medical ethics and law, communication skills, and the like, by continuously reviewing the educational programs of medical sciences and the quality of education at various universities and educational centers. In addition, while reflecting the results of its monitoring, it must follow up on the elimination of shortcomings in the educational programs of medical science students through the Ministry of Health, Treatment and Medical Education.

Article 44. Members of the HCPs have the right to continue to receive the necessary training to keep their knowledge up-to-date after graduation and formal membership in the profession. The Iran Medical Council is obligated to ensure, through appropriate means, that adequate and high-quality in-service training is provided by relevant institutions and, when necessary, to provide this training directly. Also, by continuously reviewing the quality of training and trying to eliminate its shortcomings, it should also pay attention to the adequacy and validity of training when issuing a license or professional qualification. Providing this training is of particular importance in times such as infectious disease epidemics or emerging diseases for which there is no known treatment and members of the HCPs need to receive the latest scientific achievements and research findings more than ever.

Article 45. Members of the HCPs have the right to access up-to-date sources of knowledge they need, in accordance with global standards and based on the needs and conditions of the community; therefore, relevant institutions must provide this access and the necessary infrastructure for it, including efficient and secure internet access. The Iran Medical Council is obligated to follow up on this access and facilitate it as much as possible.

Article 46. Members of the HCPs have the right to receive educational information and analysis of common medical complaints or errors and their causes and contexts from institutions that handle medical errors. Since providing this information to members of the profession helps reduce medical complaints and errors, the Iran Medical Council is obligated to take the necessary measures, including conducting analytical research, publishing extracted cases from the files reviewed by the organization anonymously while preserving the privacy of the file's stakeholders, and asking other investigating institutions, including the Forensic Medicine Organization, to make educational samples available to members of the profession.

Article 47. Members of the HCPs have the right to know the scope of their authorized interventions and to be fully aware of the description of their duties and the similarities and differences between their field of work and other professional fields. The Iran Medical Council is obligated, in cooperation with the Ministry of Health, Treatment and Medical Education and other relevant authorities, while

identifying potential areas of ambiguity, to take steps to resolve ambiguities and define professional boundaries. In this regard, it is necessary to consider issues such as avoiding monopolies, providing quality services, and improving people's access to health services.

Article 48. All members of the HCPs have the right to fair educational, training, and research opportunities. In this regard, the Iran Medical Council is obligated to investigate areas of educational discrimination for members of the HCPs, including those admission quotas that are considered unjustified, through responsible institutions such as the Supreme Council for the Cultural Revolution, the Islamic Consultative Assembly, and especially the Ministry of Health and Medical Education, it should interact and strive to eliminate educational injustice and discrimination.

Chapter Five: The Transparency and the Right to Participate in Determining one's Destiny

Article 49. Members of the HCPs have the right to express their objections, in cases such as the enactment of laws and regulations that, for any reason, they consider to be contrary to professional interests or public health and the rights and interests of their patients, through various means and within the framework of the laws of the country. Any protest, including peaceful assembly, is considered a right of members of the HCPs if it does not threaten the rights and interests of patients in any way. The Iran Medical Council is obligated to provide an appropriate mechanism for registering and holding legal protests.

Article 50. Members of the HCPs have the right to participate, directly or through their representatives in the Iran Medical Councilor scientific and professional associations, in decision-making in the field of health and matters that directly concern them. Participation in major decision-making by the Ministry of Health, Treatment and Medical Education, the Supreme Insurance Council, the approval of relevant laws in the Islamic Consultative Assembly, the approval of relevant regulations in the Cabinet, various departments of the judiciary, and crisis response headquarters are examples of how this right of theirs must be properly pursued and fulfilled. The Iran Medical Council is obligated to systematically and actively participate in decision-making by identifying influential decision-making positions and, wherever possible, utilize the capacity of its members and other institutions that represent the HCPs.

Article 51. Members of the HCPs have the right to freely participate as volunteers or voters in the elections of their respective professional, scientific, and trade union institutions, especially the elections of the Iran Medical Council, scientific associations, and the like. Any extralegal restriction on the right to elect members of the profession is against the interests of the health system and must be dealt with appropriately.

Article 52. Members of the HCPs have the right to access the annual performance report and financial statements of the Iran Medical Council and other institutions of their choice. The Iran Medical Council is required to publish its balance sheet, financial statements, and performance report annually and in a completely transparent manner and make it available to members.

Article 53. Members of the HCPs have the right to strong and efficient professional, trade, and scientific associations; therefore, it is necessary for the licensing authorities of such associations to pave the way for the establishment and operation of such institutions, based on a specific procedure developed with the participation of representatives of the HCPs. The Iran Medical Council is obligated to provide a framework for close and ongoing communication with medical group associations and help empower them. In the meantime, utilizing the diverse capacities of the associations, especially in the field of scientific advancement of the HCPs and professional self-regulation and dealing with cases of violations of professional ethics, can be considered.

Article 54. Members of the HCPs have the right to be informed of the content of the official discussions of the General Assembly and the Supreme Council of the Medical Council. To this end, it is necessary that after each meeting of these organs of the organization, the details of the discussions are documented in writing and, after literary editing and removal of any personal information that may have been raised, are made available to all members through the organization's Internet portal. In cases where, for valid reasons, it is necessary to hold a meeting in secret or where a topic is confidential, a report of the meeting must be made available to the general membership, stating the reasons for the confidentiality of the meeting or the confidential topic raised.

Chapter Six: The Right to Independence in Professional Decision-Making

Article 55. Members of the HCPs have the right to refuse to provide a health service, such as therapeutic abortion, in cases where the service in question, despite being legal, conflicts with their beliefs and convictions, provided that their refusal does not threaten the health of the patient or patients. The Iran Medical Council is required to prepare a list of cases in which refusal by members of the HCPs to perform interventions is morally and legally justified, and after approval by the Supreme Council of the Medical Council, add it to the "General Guide to Professional Ethics for Medical and Allied Professionals." Conscientious objection is prohibited in cases that have not been approved by the Iran Medical Council, and the consequences arising from it are the responsibility of the person refusing.

Article 56. All members of the HCPs have the right to act in accordance with their professional, ethical, and technical standards and should not be pressured or persecuted for insisting on these standards. The Iran Medical Council is obligated to protect the professional independence of all its members, especially those who are more vulnerable to violations of professional independence for reasons such as employment in certain institutions.

Article 57. Members of the HCPs have the right to refuse to participate in the punishment of criminals. In such cases, the person refusing to participate in the punishment should not be pressured or harassed in any way. The Iran Medical Council is obligated to defend its members in case of pressure on them in such cases.

Article 58. Members of the HCPs have the right to terminate their therapeutic relationship with a patient for technical or logical reasons or when unmanageable differences arise with the patient or their companions, in cases where initiating or continuing the therapeutic relationship would likely cause harm to one or both parties, and on the other hand, severing the relationship would not cause serious harm to the patient and the patient's treatment could be continued by another available professional. In such cases, it is necessary to record the details of the matter in the patient's file, stating the reasons, and provide the patient with a copy of the patient's file, along with the necessary education and information to continue treatment.

Article 59. Members of the HCPs have the right to make independent decisions in the diagnosis, treatment, and provision of healthcare services, free from external pressures. In this context, any coercion against professionals (including forced hospitalization of individuals without medical justification, or pressure to prescribe unnecessary services aimed at increasing the revenue of healthcare institutions) is strictly prohibited. The Iranian Medical Council is obligated to investigate such cases through diverse methods and by collaborating with relevant stakeholders to safeguard practitioners against these pressures.

Table1 - Extracted Themes, Categories, and Subcategories

Main Theme	Categories	Subcategories
**The right to enjoy fundamental rights**	***Right to a decent income***	Fixed and predictable income
		Fair wage
		Increased payments
		Sufficient income
	Social acceptability	Respect from colleagues
		Respect from society
	Job security	Improved working conditions
		Job opportunities
		Autonomy in treatment
	Work justice	Elimination of inequalities
		Educational justice
	Right to protest	Public gathering
		Professional protest
**Accountability of the Ministry of Health**	***Right to professional development***	Expansion of scientific boundaries
		Continued education
	Improvement of academic system and right to quality medical education	Proper educational curricula
		Development of necessary educational infrastructure
	Backbone of the medical community	Physical support

		Social support
		Professional support
	Holistic approach in lawmaking	Transparency and fairness in legislation
		Information dissemination
		Referral system
**Accountability of the Medical Council**	***Enhancement of professional efficiency***	Involvement in medical lawmaking
		Supervisory role
		Establishment of strong and efficient unions
		Raising awareness
		Revision of medical tariffs
		Reform of physicians' tax system
	Improvement of support functions	Mental and psychological support
		Legal support
		Physical support
		Welfare support
	Education	Public awareness
		Legal education for doctors
		Continuing education
	Smart revision of legal issues	Establishment of a dedicated legal structure for physicians
		Judicial law reforms

**Reformation of the Insurance System**	***Improvement of insurer organizations' performance***	Deductions
		Timely settlement of claims
		Fair insurance payments
		Insurance decision-making
		Reduced physician insurance tariffs
	Insurance coverage	Professional liability insurance
		Retirement insurance
**Special Support and Attention to Specific Stages**	***Residents***	Standardized working hours
		Financial and welfare facilities
		Improved professional environment
		Educational opportunities in hospitals
		Health and liability insurance benefits
	General practitioners	Job opportunities
		Increased income
		Increased social capital
